# A Hypothesis Concerning a Potential Involvement of Ceramide in Apoptosis and Acantholysis Induced by Pemphigus Autoantibodies

**DOI:** 10.1155/2010/702409

**Published:** 2010-05-18

**Authors:** Wendy B. Bollag

**Affiliations:** ^1^Charlie Norwood VA Medical Center, Research Service, One Freedom Way, Augusta, GA 30904, USA; ^2^Departments of Physiology, Medicine, Orthopaedic Surgery, Cell Biology, and Anatomy, Medical College of Georgia, 1120 15th Street, Augusta, GA 30912, USA

## Abstract

Autoimmune diseases affect more than 50 million Americans, resulting in significant healthcare costs. Most autoimmune diseases occur sporadically; however, endemic pemphigus foliaceus (EPF) is an autoimmune skin disease localized to specific geographic loci. EPF, and the related diseases pemphigus vulgaris (PV) and pemphigus foliaceus (PF), are characterized by skin lesions and autoantibodies to molecules found on epidermal keratinocytes. A variant of EPF in patients from El Bagre, Colombia, South America, has recently been reported to be distinct from previously described loci in Brazil and Tunisia epidemiologically and immunologically. As in PF and EPF, El Bagre EPF patients exhibit autoantibodies towards desmoglein-1, a cell adhesion molecule critical for maintaining epidermal integrity. An association of El Bagre EPF with sun exposure has been detected, and ultraviolet irradiation also exacerbates symptoms in PV, PF and EPF. Our hypothesis is that: (1) the autoantibodies generate pathology through an alteration in ceramide metabolism in targeted keratinocytes, resulting in apoptosis and/or cell death and acantholysis, but only when the cell's ability to metabolize ceramide is exceeded, and (2) apoptosis in response to this altered ceramide metabolism is initiated and/or exacerbated by other agents that increase ceramide levels, such as cytokines, ultraviolet irradiation, and senescence.

## 1. Introduction

More than 50 million Americans suffer from autoimmune diseases. Because of the chronic nature of this group of diseases, their treatment results in a tremendous cost to healthcare as well as to serious reductions in the quality of life of affected individuals. Pemphigus refers to a class of rare autoimmune skin diseases characterized by epithelial blistering and acantholysis. Pemphigus vulgaris (PV) blisters occur in mucosal tissues and the skin whereas the lesions of pemphigus foliaceus (PF) exhibit a localization to the suprabasal epidermis. The main pathogenic PV autoantibody recognizes desmoglein—(Dsg) 3, a desmosomal cadherin expressed in mucosae and the epidermis. PF antibodies are predominantly directed towards Dsg-1, another cell adhesion molecule critical for the maintenance of epidermal integrity. Most autoimmune diseases, including PV and PF, occur sporadically and are widely scattered geographically. However, endemic pemphigus foliaceus (EPF) represents an autoimmune disorder that is limited to a well-defined geographic area (reviewed in [1, 2]), such as Brazil or, as reviewed below, El Bagre, Columbia in South America [3]. This El Bagre EPF is also characterized by acantholytic skin lesions and by autoantibodies to Dsg-1 [3–5]. However, the mechanism by which the autoantibodies found in the sera of patients with El Bagre EPF, as well as with PV, PF, and EPF, result in the blistering skin lesions typical of pemphigus is largely unknown.

## 2. El Bagre Endemic Pemphigus Foliaceus (EPF)

A novel variant of EPF in patients from an area around E1 Bagre, Colombia, South America was recently identified by Abreu-Velez and colleagues [3]. This focus of EPF is distinct from previously described EPF foci in Brazil and Tunisia both epidemiologically and immunologically. Thus, patients with El Bagre EPF are typically men aged from forty to sixty (with a few postmenopausal women), and symptoms often resemble those of paraneoplastic pemphigus, but without the accompanying malignancy [3]. The autoantibody profile of these El Bagre EPF patients is distinct from that of patients with the Brazilian form of EPF, also known as fogo selvagem (see below). As with other EPF foci as well as PF, the El Bagre disease is characterized by skin lesions with hyperkeratosis, acanthosis, and acantholysis. Immunofluorescence studies indicate that the sera of these patients possess autoantibodies that recognize an antigen(s) on the keratinocyte cell surface producing typical intercellular staining in the epidermis [4]. Approximately two-thirds of the E1 Bagre EPF patients exhibit a form localized to the skin; however, one-third develop a more severe form characterized by systemic symptoms resembling lupus. There also appears to be a genetic component to the development of this disease, since certain ethnic groups show a predisposition to acquire E1 Bagre EPF. On the other hand, the involvement of environmental factors in the development of El Bagre EPF is suggested by the restricted geography (this EPF is limited to individuals living in the area surrounding E1 Bagre) as well as the fact that some patients have converted from the systemic to the localized form of the disease after moving from the area [3]. Additional evidence of an environmental parameter is the finding of a strong association between the amount of sun exposure and the development of the disease [3]. However, the precipitating factor(s) that triggers the disease is unknown.

## 3. Immunologic Features of El Bagre EPF

Indirect immunofluorescence (with El Bagre EPF sera) on human skin sections revealed intercellular staining in all patients, with some EPF sera also showing reactivity with the basement membrane zone [4]. Approximately 10% of the control individuals from within, but not from outside, the endemic area also demonstrated intercellular immunoreactivity. Immunoprecipitation and immunoblotting analyses indicated that the antigens recognized by the EPF autoantibodies comigrated (by gel electrophoresis) with Dsg-1, desmoplakin, envoplakin, and periplakin; and studies using baculovirus-expressed desmosome-associated proteins showed that these patients possess antibodies recognizing Dsg-1, envoplakin, and periplakin [6]. Dsg-1 represents an important antigen for the autoantibodies of patients with fogo selvagem [7] as well; however, this study [6] demonstrated a distinct immunoreactivity profile in El Bagre EPF versus fogo selvagem. These results again point to the idea that El Bagre EPF represents a novel variant of the disease. Nevertheless, the mechanism by which recognition of Dsg-1, and perhaps other antigens, contributes to the observed apoptosis [8, 9] and development of lesions in EPF is not known [1].

## 4. Pemphigus and Apoptosis, Apoptolysis and Oncosis

Although it is appreciated that there is significant apoptosis in pemphigus, there has been controversy concerning whether or not apoptosis precedes and/or is required for acantholysis (reviewed in [10]). Recent evidence suggests that while other processes, such as oncosis [11], cell shrinkage, and a process that has been termed apoptolysis (reviewed in [12]), play a role in the development of skin lesions, apoptotic cell death also clearly contributes to acantholysis. Indeed, many investigators have reported the ability of inhibitors of apoptosis-activated caspases to inhibit acantholysis both *in vitro* and *in vivo*, in a passive transfer mouse model of pemphigus ([11] and reviewed in [12]). On the other hand, there seems to be some differences in the mechanism of cell death induced by autoantibodies from different patients. Thus, Grando and colleagues [11] found two subsets of PV autoantibodies: for one subset, cell death seemed to be primarily initiated by caspase-dependent processes (although calpain was also involved), and in the other, calpain initiated cell death (although caspases also contributed). 

**Figure 1:**
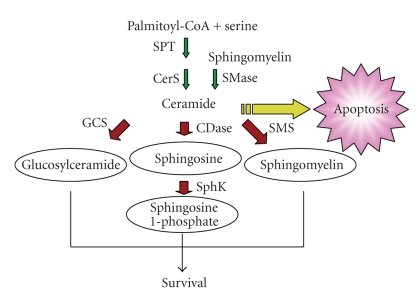
Ceramide Metabolism. Ceramide is produced *de novo* via serine palmitoyltransferase (SPT) and ceramide synthase (CerS), or from sphingomyelin via sphingomyelinase (SMase), and induces apoptosis. In turn, ceramide is metabolized via multiple mechanisms, including glucosylceramide synthase (GCS), ceramidase (CDase) sphingosine kinase (SphK), and sphingomyelin synthase (SMS), with this metabolism of ceramide allowing cell survival.

Data in the literature indicate that pemphigus sera are able to trigger lipid metabolism in treated keratinocytes. Based on an in-depth understanding of ceramide and sphingolipid metabolism and the role of ceramide as a mediator of apoptosis in multiple cell systems (reviewed in [13, 14]) as well as data in the literature, we hypothesize that pemphigus autoantibodies contribute to lesional pathology by altering sphingomyelin metabolism to result in elevated ceramide levels that trigger cell death via apoptosis (Figure 1). Furthermore, we hypothesize that this process is exacerbated in keratinocytes exposed to other agents that affect ceramide metabolism, such as cytokines and ultraviolet (UV) irradiation (see below). This hypothesis represents a novel interpretation of the data and could potentially provide a mechanism by which autoantibodies lead to cell death and apoptosis, oncosis and apoptolysis.

## 5. Ceramide As an Antiproliferative, Proapoptotic Signal

Ceramides are well-known structural components of the skin and help to form the water-permeability barrier of the epidermis (reviewed in [15]). However, ceramide can also function as a lipid second messenger. Approximately two decades ago, investigators began reporting on the ability of certain cytokines and other signals to activate sphingomyelinase, an enzyme that hydrolyzes sphingomyelin to form ceramide and phosphorylcholine (reviewed in [16]). In addition, using synthetic ceramides to mimic the effects of sphingomyelinase-activating agents, researchers demonstrated the ability of this lipid to function as a signaling molecule, mediating a number of important effects. Other studies have shown that ceramide can be generated by a *de novo *pathway initiated by serine palmitoyltransferase as well (Figure 1), for instance, in response to chemotherapeutic agents (reviewed in [13]). Most often, these increases in ceramide are associated with growth arrest, differentiation, senescence, and apoptosis, as has been shown in numerous cell types (reviewed in [17]), including keratinocytes [18, 19]. In particular, ceramide produced in response to a number of cell stresses has been shown to trigger apoptosis (reviewed in [17]).

**Figure 2:**
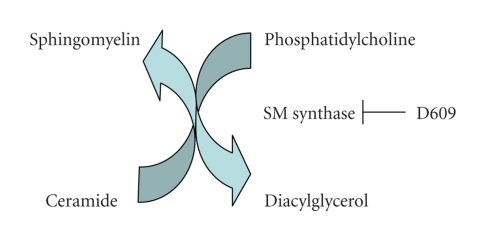
Sphingomyelin synthase. The reaction catalyzed by sphingomyelin synthase (SM synthase) results in a reduction in ceramide and an increase in diacylglycerol and sphingomyelin levels. The reported phosphatidylcholine-specific phospholipase C inhibitor D609 also inhibits sphingomyelin synthase activity.

Interestingly, hyperproliferative cancer cells are often able to evade the apoptosis triggered by agents that increase ceramide by utilizing multiple metabolic enzymes to decrease ceramide below critical levels (reviewed in [20]). As an example, in multidrug-resistant cells the enzyme glucosylceramide synthase (GCS), which glycosylates the ceramide induced by chemotherapeutic agents to produce glucosylceramide (Figure 1 and reviewed in [11]), is elevated. Overexpression of GCS converts drug-sensitive cells to resistant ones, whereas decreasing GCS levels with antisense constructs changes resistant cells to a sensitive phenotype [21, 22]. This same enzyme also appears to protect keratinocytes against ceramide-induced stress/apoptosis by converting ceramide to glucosylceramide [23]. Similarly, virally transformed cells (SV40-transformed human lung fibroblasts) exhibit increased sphingomyelin synthase activity [24]. Sphingomyelin synthase transfers the choline headgroup from phosphatidylcholine to ceramide to generate sphingomyelin and diacylglycerol (Figure 2), thereby decreasing ceramide levels. Ceramide can also be metabolized by ceramidase, which hydrolyzes ceramide to sphingosine and is activated, for instance, by growth factors (e.g., [25]). In general, sphingosine is antiproliferative and/or proapoptotic, that is, induces similar effects to ceramide (reviewed in [26]). On the other hand, sphingosine can be phosphorylated to yield sphingosine 1-phosphate (S1P) by sphingosine kinase (of which there are two identified isoforms, sphingosine kinase-1 and -2). S1P can act as a first messenger by binding to a family of GTP-binding protein-coupled receptors, the S1P receptors, but may also signal intracellularly (reviewed in [27]). In most cases, S1P mediates proliferative cell responses and/or cell survival [27], with the combined activities of ceramidase and sphingosine kinase possibly accounting for the observation that in some cell types certain ceramide-elevating agents increase proliferation. Thus, any agent that increases ceramide levels might be expected to activate one or more ceramide metabolic pathways as the cell strives to reduce ceramide and protect itself from the apoptotic effects of this lipid signal. Indeed, 1,25-dihydroxyvitamin D_3_-stimulated sphingomyelinase activation, which would be expected to increase ceramide production, appears not to induce apoptosis because of the concominant generation of S1P [28], which in human keratinocytes S1P is reported to be prodifferentiative (e.g., [29]). In contrast, if ceramide levels rise beyond a critical threshold, that is, above the levels that can be metabolized by the various ceramide metabolic pathways apoptosis might be anticipated to result.

## 6. Pemphigus and Ceramide

Seishima et al. [30] investigated the effect of PV autoantibodies on signaling processes in DJM-1 cells, a squamous cell carcinoma cell line. These authors found that addition of immunoglobulins from pemphigus patients increased the levels of diacylglycerol derived from phosphatidylcholine. This increase was not the result of activation of phosphatidylcholine-hydrolyzing phospholipase D, resulted in the release of phosphorylcholine and could be inhibited by D609. Furthermore, preadsorption of the immunoglobulins with Dsg-1 and -3 prevented the signaling effect [30]. Stanley and colleagues have suggested that autoantibodies to either Dsg-1 or -3 can produce blisters in pemphigus diseases, with the location of the lesions dependent on the distribution of these two molecules in various epithelia [31]. Thus, antibodies directed at either of these desmosomal components should induce similar effects, and anti-Dsg antibodies present in PV, PF, or EPF, including El Bagre EPF, would be expected to trigger the same signaling events as those elicited by the autoantibodies in PV sera. Seishima et al. suggested that the pemphigus autoantibodies activate a phosphatidylcholine-specific phospholipase C [30]; however, no such enzyme has as yet been purified and/or cloned in mammals. In addition, it has been shown that D609 inhibits sphingomyelin synthase [24, 32] in addition to (or instead of) the reported phosphatidylcholine-specific phospholipase C. As mentioned previously, sphingomyelin synthase transfers the choline headgroup from phosphatidylcholine to ceramide to form diacylglycerol and sphingomyelin (see above and Figure 2). Because phosphatidylcholine is “consumed” and diacylglycerol is generated by this enzyme, it is difficult to distinguish between the activity of sphingomyelin synthase and that of a hypothetical phosphatidylcholine-specific phospholipase C without monitoring sphingolipid metabolism. Thus, the idea that the pemphigus autoantibodies activate a sphingomyelinase to generate ceramide (and release phosphorylcholine), and this ceramide is metabolized by sphingomyelin synthase (to decrease phosphatidylcholine and increase diacylglycerol) is consistent with the observed findings. 

The possible involvement of ceramide in the pathology of the skin lesions is also consistent with the observed association of El Bagre EPF disease with certain environmental factors such as sun exposure [3]. Sun exposure has also been reported as an exacerbating factor in PV, PF, and fogo selvagem (e.g., [33, 34]) (see below). UV irradiation is known to raise ceramide levels in keratinocytes [19, 35], as do many cell stresses in other cell types (reviewed in [36]), suggesting that the autoantibodies in and of themselves may not be pathogenic without some additional perturbation of the cells. Indeed, relatives of the El Bagre EPF patients have been observed to possess immunoreactivity to EPF antigens, as measured by immunoprecipitation, immunoblotting [5, 6], and an enzyme-linked immunosorbent assay (ELISA) [37], yet these individuals show no evidence of disease [3, 37]. A similar phenomenon has been observed in fogo selvagem (Brazilian EPF) [38], and, in addition, fogo selvagem autoantibodies are also known to bind to oral mucosa but do not induce lesion formation [39]. On the other hand, cytokines, such as tumor necrosis factor-alpha (TNF-*α*), interleukin-1 (IL-1), and interferon gamma (IFN*γ*), are all known to elevate ceramide levels ([40] and reviewed in [41, 42]) and are present in the lesions of autoimmune skin diseases [43–45]. Thus, environmental factors that elevate the quantities of these agents may contribute to the cell perturbations that result in pathology of the autoantibodies. Indeed, Puviani et al. [8] have demonstrated that pemphigus sera contain high levels of Fas ligand (FasL). FasL is also a well-known activator of ceramide production through sphingomyelinase activation (reviewed in [41]) and may stress the keratinocytes sufficiently that they may be unable to metabolize the ceramide below apoptotic levels. If ceramide cannot be reduced below certain levels, the keratinocyte would be triggered to undergo apoptosis, as has been seen in anti-FasL-treated cultured keratinocytes [46] and in acantholytic lesions in pemphigus (reviewed in [10]). Finally, Wang et al. [47] have demonstrated that replicative senescence enhances the apoptotic effect of pemphigus autoantibodies. Ceramide levels are known to be elevated in senescence [48, 49], providing another link between ceramide and apoptotic cell death in pemphigus. Thus, we hypothesize that the autoantibodies, and perhaps FasL [8, 50], and/or other cytokines, present in pemphigus sera increase ceramide levels and in conjunction with other cell stresses, such as sun exposure and age, raise ceramide sufficiently to trigger apoptosis.

## 7. Possible Mechanisms of Ceramide Action

The question remains: how does ceramide act as a signal to induce apoptosis and other cellular responses? As this lipid second messenger is further studied, it has become apparent that it affects multiple enzymes and proteins, both directly and indirectly, to exert its effects. As an example, ceramide and ceramide-stimulating stresses are known to activate the mitogen-activated protein kinase, p38 (e.g., [51–53]). Activation of p38 has also been observed in keratinocytes upon treatment with pemphigus sera [12], providing another possible link between pemphigus and ceramide. Ceramide has also been shown to activate both a protein phosphatase and a protein kinase, the so-called ceramide-activated protein kinase identified as kinase suppressor of ras (ksr) (reviewed in [13]). In addition, ceramide, or its phosphorylated metabolite ceramide 1-phosphate, appears to modulate the activity of enzymes involved in alternative splicing to yield differential splicing of certain gene products such as caspase-9 (a proapoptotic enzyme) and Bcl-xL (an antiapoptotic mediator) [54]. The result is greater levels of the proapoptotic splice forms of these proteins (Bcl-xS and caspase-9L) [54]. Ceramide also affects multiple proteins involved in the apoptotic pathway, including Akt (an antiapoptotic, prosurvival protein that is inhibited by ceramide action) and cathepsin D, a lysosomal protease involved in apoptosis, among others (reviewed in [55]). In addition, ceramide inhibits mitochondrial function [14], thereby enhancing mitochondrial pathway-mediated apoptosis (reviewed in [14]). Thus, ceramide functions through multiple mechanisms as a proapoptotic cell signal in many cell types including keratinocytes. In addition, ceramide and its metabolites also likely play a role in inflammation (reviewed in [56]).

Although ceramide seems to function generally as an antiproliferative and/or antisurvival (proapoptotic) signaling molecule, there is emerging evidence that the cellular localization of the generated ceramide may affect the ultimate cell response (reviewed in [17, 57]). For instance, overexpression of a bacterial SMase in mitochondria, but not the cytoplasm, Golgi apparatus, endoplasmic reticulum, plasma membrane, or nucleus, triggers apoptosis in MCF7 breast cancer cells [58]. In addition to effects of compartmentalization, different ceramide species, possessing diverse fatty acids from 16 to 24 carbons in length, appear to have different signaling roles. There are five ceramide synthase isoforms, CerS1-5, which have distinct specificities for the formation of various ceramide species with different carbon lengths (reviewed in [17]). Knock down of CerS6, which produces 16 carbon-containing ceramide (C16-ceramide), induces endoplasmic reticulum stress-induced apoptosis, and overexpression of this enzyme protects against ER stress and promotes squamous cell carcinoma tumor growth *in vivo* [59]. On the other hand, overexpression of CerS1 (forming C-18 ceramide) inhibits tumor formation, indicating that not only the site of ceramide production but also the species generated is important.

## 8. Hypothesis

**Figure 3:**
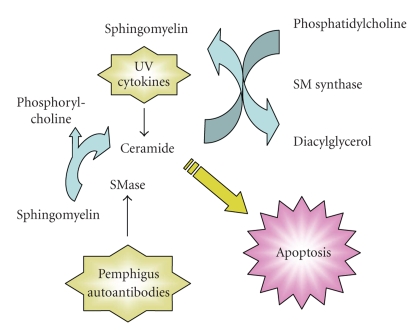
Hypothesized Involvement of Sphingolipid Metabolism in the Keratinocyte Response to Pemphigus Autoantibodies. Our hypothesis is that pemphigus autoantibodies binding to Dsgs results in the activation of sphingomyelinase (SMase) to produce ceramide and phosphorylcholine. In an attempt to survive, keratinocytes activate sphingomyelin synthase (SM synthase). The result is an increase in phosphorylcholine and diacylglycerol levels and a reduction in phosphatidylcholine. Stimuli that overwhelm the ability of the cell to metabolize ceramide, such as cytokines or ultraviolet (UV) light (or senescence), which increase ceramide levels, are proposed to allow manifestation of pathologic skin lesions. Note that UV light increases ceramide in at least two ways: first, by activation of both SMase-mediated [35] and *de novo* ceramide production [19] and second, by decreasing ceramidase activity/expression [60]. Ceramidases are key in the response of keratinocytes to UV light because (1) they metabolize ceramide and decrease its levels and (2) they generate sphingosine, which can be converted to S1P (which allows cell survival) by the action of sphingosine kinase. Indeed, knocking down sphingosine kinase sensitizes keratinocytes to UV-induced apoptosis [60]. In addition, ceramide and its metabolites can activate cytosolic phospholipase A2 and cyclooxygenase-2, suggesting that eicosanoids might be elevated and potentially contribute to the pemphigus disease process (reviewed in [56]).

We propose the idea that pemphigus autoantibodies, upon binding to Dsgs, trigger sphingomyelin hydrolysis accompanied by a corresponding stimulation of sphingomyelin synthase activity (Figure 3), as the cell attempts to reduce proapoptotic ceramide amounts. If the cell is efficient enough, no change in ceramide or sphingomyelin levels may occur, although a reduction in phosphatidylcholine levels and an increase in phosphorylcholine release and diacyl-glycerol quantities would be expected. In this case, acantholysis and other hallmarks of pemphigus would not be observed despite the ability of the antisera to bind Dsgs. Indeed, this need for interaction between the binding of autoantibodies and additional changes in ceramide metabolism may be one explanation for the low levels of anti-Dsg antibodies without concomitant disease seen in some individuals in the El Bagre area [37] and fogo selvagem [61], as well as non-pathogenic antibodies observed in some pemphigus patients (reviewed in [62]). We also hypothesize that coincubation of keratinocytes with pemphigus sera and cytokines, such as FasL, or the combination of pemphigus sera and irradiation with UV light, will result in enhanced levels of ceramide relative to any of the agents alone. Since these combinations would be expected to overwhelm the ability of the cell to metabolize the pemphigus sera-induced production of ceramide, we would also anticipate that cytokines and UV light will act synergistically with pemphigus autoantibodies to stimulate markers of apoptosis and cell death. Consistent with this idea, inhibition of the action of some cytokines, in particular FasL and TNF-*α*, can inhibit acantholysis in experimental models of pemphigus (reviewed in [12]), and a beneficial action of new TNF-*α* blocking biological agents in patients has also been reported [63, 64]. In addition, ultraviolet irradiation is known to worsen pemphigus [11]. Similarly, there is a clear association between sunlight exposure and El Bagre EPF [3]. The idea that UV induces ceramide production that is at least partially compensated for by an increase in ceramide clearance is supported by the findings of Uchida and colleagues, who reported (in abstract form) that both knock down of ceramidase levels [60] and incubation with N-acyl-ethanolamine (NAE) compounds that inhibit acidic and neutral ceramidases sensitize keratinocytes to UV-induced apoptosis [65]. These authors also showed that inhibition of the NAE-hydrolyzing enzymes, NAE amidohydrolase, fatty acid amidohydrolase, and NAE-hydrolyzing acid amidase, further exacerbates the effects of UV irradiation. NAE compounds occur naturally and increase with cell stresses including UV exposure and xenotoxics; thus, enhanced production of NAEs, as well as decreased ceramidase activity/expression in response to UV, could significantly impair ceramide clearance [65]. It should be noted that the epidermis has been shown to express all five known ceramidase isoforms, with some localized to the basal layer and others to the differentiating compartments [66]. In addition, alkaline (aCER1) and acid (AC) ceramidases are upregulated during elevated extracellular calcium-induced keratinocyte differentiation and appear to mediate the differentiative effects of calcium [67]. Thus, ceramidases are clearly vital to epidermal physiology and perhaps also pathophysiology, likely by both decreasing ceramide levels and providing sphingosine for production of S1P [60].

On the other hand, it is possible that pemphigus sera do not activate sphingomyelinase but rather stimulate the activity of sphingomyelin synthase through the ligation of cell adhesion molecules. It is widely recognized that cell adhesion can elicit signal transduction processes (reviewed in [68]). Perhaps antibody binding of Dsgs (or possibly other cell surface proteins recognized by pemphigus autoantibodies) results in stimulation of signaling and activation of sphingomyelin synthase. This result would then predict that antibody-mediated loss of the Dsg (or other cell surface proteins) would inhibit this signal, again leading to apoptosis, either through elevations in ceramide levels or via reductions in protein kinase C-activating diacylglycerol. Diacylglycerol appears to be a prosurvival, antiapoptotic signal in most cell types (reviewed in [13]), presumably in part through its ability to activate sphingosine kinase and stimulate the production of S1P from ceramide [69]. Pemphigus sera have also been reported to stimulate phosphoinositide hydrolysis [70], which would result in the generation of diacylglycerol. Alternatively, it is possible that antibody-mediated loss of Dsg function (rather than of the protein itself) may mediate the changes in cell signaling processes that lead to blister formation in pemphigus. However, this possibility is argued against by recent evidence suggesting that Dsg-1 is proapoptotic, such that RNA interference to decrease Dsg-1 protects keratinocytes against ultraviolet irradiation-mediated apoptosis [71]. In any case, these ideas should be testable by experiments to examine the effect of pemphigus sera on the levels of ceramide and other sphingolipids in keratinocytes, and research into the role of sphingolipid metabolism in the keratinocyte acantholytic response to pemphigus autoantibodies seems warranted.
